# Open questions on the interaction dynamics of molecules and clusters in the gas phase

**DOI:** 10.1038/s42004-022-00646-y

**Published:** 2022-03-08

**Authors:** Michael Gatchell, Henning Zettergren

**Affiliations:** 1 Department of Physics, Stockholm University, 106 91 Stockholm, Sweden; 2 Institut für Ionenphysik und Angewandte Physik, Universität Innsbruck, Technikerstr. 25, A-6020 Innsbruck, Austria

**Keywords:** Chemical physics, Reaction kinetics and dynamics

## Abstract

Emerging experimental techniques combined with theoretical advances allow unprecedented studies of the dynamics of gas phase molecules and clusters induced in interactions with photons, electrons, or heavy particles. Here, the authors highlight recent advances, key open questions, and challenges in this field of research with focus on experimental studies of dynamics of ions stored on millisecond timescales and beyond, and its applications in astrochemistry and astronomy.

When gas phase molecules or clusters interact with photons, electrons, or heavy particles, the electronic and structural dynamics set in motion may extend on timescales spanning twenty orders of magnitude—from attoseconds to minutes—while their effects may continue being felt on astronomical timescales (Fig. 1). The timescales of these dynamics span those typical of the electronic motion and its coupling to nuclear motion and molecular rearrangements, to those for ultraslow electron emission, radiative cooling (vibrational and rotational relaxation), and fragmentation. This means that an arsenal of complementary state-of-the-art experimental, computational and theoretical tools is required to fully unravel the dynamics, and to answer open key questions such as:

- How do molecules form and survive in the interstellar medium?
- What are the conditions for the building blocks of life to evolve in extraterrestrial environments?
- What are the mechanisms behind aerosol formation in planetary atmospheres?

**Fig. 1:**
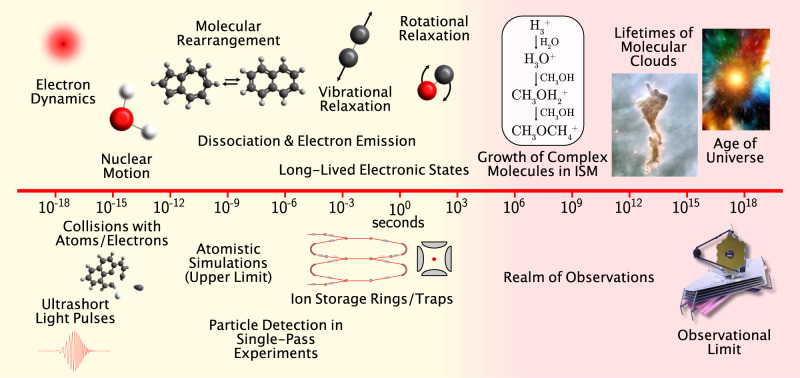
Gas phase dynamics take place over many orders of magnitude in time. Molecular excitation can be induced by ultrafast processes such as short light pulses or fast ion collisions, and the resulting dynamics may proceed for minutes or longer. The figure shows typical time domains where different processes dominate the dynamics. Recent developments in cryogenic ion beam storage allow for time-resolved studies of these processes for longer than ever before possible, up to minutes or hours (shaded yellow). Gas phase dynamics play an important role in interstellar space where the relevant timescales can be millions of years or more (shaded red).

A complete description of the intriguing developments being made in this rapidly growing field is unfortunately beyond the scope of this short comment (see, e.g., recent topical roadmaps^1–4^ and references therein for more details). Here, we focus on the status of some recent advances while sharing our view of a selection of common challenges, as well as more specific ones, and key questions to advance the understanding of the dynamics of ions extending to ultralong timescales.

## Status and recent advances

The development of novel light sources such as attosecond lasers and X-ray free electron lasers (XFELs) have opened up completely new research opportunities towards real-time monitoring of electronic dynamics, electron emission, charge transfer, photon emission, and the breaking and making of molecular bonds^2,5^. In parallel to these advances, studies using ingenious probe and detection schemes at large scale synchrotron facilities or with lab-based table-top lasers have been paramount to further the understanding of ultrafast relaxation processes^1,2^, as well as slower dynamics extending up to microseconds^1,2^. The latter often make use of advanced mass spectrometry techniques combined with multi-coincidence detection. Similar techniques are also commonly used in experiments where dynamics is ignited in interactions with electrons or atoms/molecules/clusters (neutrals or ions)^3,4^, and have been instrumental to advance the understanding of, e.g., fragmentation dynamics, intracluster reactions, and the ergodicity of such processes. Here, a key question that has been addressed is whether the total excitation energy alone, or also the way energy is deposited in the interactions, dictates the subsequent dynamics. It has, for instance, been shown that atomic projectiles have the unique possibility to knock out single atoms in molecules through billiard ball type interactions^6^. The so-formed fragments may be highly reactive and thus key intermediate steps in, e.g., molecular growth processes. Combined with photon-impact studies of the types outlined above, interaction dynamics involving electrons and heavy particles have been crucial for a new understanding of, e.g., radiation damage processes at the nanoscale, key reactions for atmospheric science, and the evolution of molecules in space.

Studying the interaction dynamics on even longer timescales requires storing the molecular and cluster ions. Recent advances in ion trapping and storage techniques designed for, e.g., specific spectroscopic applications and/or detection of certain types of reaction products enable monitoring the dynamics up to milliseconds and longer^1,7^. Building on the developments of electrostatic ion beam storage devices^8,9^, a new generation of cryogenically cooled storage rings offer unique possibilities to study interactions with internally relaxed ions and cooling dynamics in new time domains^9^. These allow for studies of, e.g., (i) charge-, mass- and energy- transfer processes in sub-eV collisions between pairs of oppositely charged ions (see, e.g., Grumer et al.^10^ and references therein), (ii) reactions between stored ions and free electrons or neutrals (atoms or molecules) that may be fine-tuned down to micro- and millielectronvolts^11^, and (iii) studies of ultraslow fragmentation, electron emission and radiative cooling (vibrational and rotational relaxation) processes up to hundreds of seconds^1,12^. In recent years there has also been a strong development of compact and versatile ion beam storage devices designed for, e.g., merged beams interactions^13^ and for providing kinematically complete information on reactions induced in interactions with a variety of projectile beams^14^.

In parallel to these experimental developments, theoretical and computational advances have been absolutely crucial for instance in the design of new experiments, analysis and interpretation of experimental results, and for input in large-scale models of, e.g., astrochemical, atmospheric, and biochemical processes (see, e.g., Zettergren et al.^1^ and references therein).

## Key open questions and challenges

The experimental advances outlined above provide new opportunities but also new challenges for experiments as well as theory. A common challenge in gas phase studies is to prepare molecules and clusters in well-defined structures and quantum states (electronic- and rotational- and vibrational energy) before the interaction dynamics is ignited. To accomplish this becomes extremely challenging with increasing molecular complexity. Furthermore, it is essential to produce sufficiently large target quantities or ion beams intense enough to study specific processes. The key here is to further refine and combine techniques in novel ways, for instance, soft ionization methods designed to bring fragile molecules into the gas phase, isomer selection schemes, pre-trapping, and cryogenic cooling of ions, as well as state-selective laser probing techniques. It is equally important and challenging to fully characterize the reaction products through, e.g., advanced pump-probe schemes, action spectroscopy, and the development of new techniques and methods to monitor neutral and charged reaction products and the emission of electrons and photons.

### Dynamics of stored ions on ultralong timescales

The experimental and theoretical advances in ion beam storage technologies offer unique possibilities towards answering key open fundamental questions, which are largely unknown for interaction dynamics involving internally cooled molecular and cluster ions:

- Which quantum states are populated in cation-anion, ion-neutral, and ion-electron reactions, and what are the corresponding reaction rates?
- How do these rates depend on the isomeric structures, the quantum state properties (rotational–vibrational energy and electronic structure), and the collision energy of the colliding partners?
- What role do these reactions play for, e.g., the ionization balance and survival of molecules in space?

Characterizing the initial quantum states in poly-atomic systems is a challenge in such studies as they generally do not cool down to their rotational–vibrational ground states when produced hot and then stored. One solution is to prepare the ions using a source that provides cold ions, for instance by using He-droplet or electrospray ionization techniques, together with a pre-trap outside the storage device that accumulates a sufficient number of ions prior to injection. Such challenging techniques are currently being developed. Another challenge is to develop techniques for detecting two or more neutral reaction products in coincidence under the most demanding (cryogenic) conditions where conventional particle detectors struggle. One of the most promising technologies is based on microcalorimetry, which will allow for determining the mass of neutral particles and not only their positions and arrival times. This is absolutely crucial for disentangling different reaction pathways and hence their branching fractions and rates. Another challenge is to detect charged reaction products having mass-to-charge ratios close to the circulating ion beams, which are typical when, e.g., complex molecules dissociate.

These advances will also lead to a detailed understanding of ultraslow dynamics occurring on millisecond timescales and beyond, and to answer key open questions such as:

- What are the rates and branching fractions for fragmentation, radiative cooling, electron emission, and isomerization when molecules and clusters are internally heated?
- How are these processes influenced by electronic, vibrational, and rotational excitations, and internal energy flow, and how do they affect the evolution of interstellar molecules?

Experimental results utilizing the new breed of cryogenic ion storage devices have shown that competing decay pathways can proceed on timescales of minutes or longer and that quantum effects play a large role in the balance between the different channels. Disentangling the different processes presents a challenge for experiment and theory alike. As an example, for atomic clusters with high rotational quantum numbers, certain decay channels may be frozen out at the same time as new ones are opened (such as tunneling), a balance that can rapidly change as the systems are followed in time^7^. The novel detection techniques described above will play an important role in advancing our understanding of these processes, as will advances in theoretical modeling. Timescales of minutes are well beyond the reach of full quantum simulations, though certain aspects can be reproduced by artificially increasing the rate of the dynamics, e.g., by increasing the simulated temperature. However, in many cases, statistical modeling of decay processes is the only viable option. As is always the case, these models are only as good as the approximations they contain, but they can offer insight into the competition between different mechanisms over long timescales^7^. Advancements in experimental capabilities are furthermore providing new possibilities for testing such models and additional motivation for developing new and improved models.

## Outlook

The dynamics of gas phase atoms and molecules is a topic of fundamental research largely belonging to chemistry and physics, but the potential applications of the processes being studied are widespread. From characterizing plasmas to understanding how radiation damages organic molecules, and from atmospheric science to astrophysics, gas phase dynamics comes in many flavors. The last example is briefly covered in Fig. 1. The evolution of the interstellar medium (ISM) is driven by the dynamics of atoms and molecules present there. A surprisingly rich inventory of chemical species—from free protons to complex organic molecules—is constantly being processed by intense radiation fields from massive stars and energetic particles from stellar winds and supernovae. The timescales of the excitation mechanisms are the same as in laboratories on earth, but the consequences of these interactions are felt on timescales spanning over many millions of years, the typical lifetime of molecular clouds in the ISM. In addition to improvements in observational techniques, such as the ongoing deployment of the James Webb Space Telescope, many advancements in our understanding of interstellar environments come from experimental and theoretical research on gas phase dynamics. Two recent examples of this are detailed studies of the destruction mechanisms of astrophysically important helium hydride ions^11^ and PAH molecules^15^ in cryogenic ion storage rings. Further experimental developments will lead to new insights into the importance of gas phase dynamics in different environments. Types of developments envisioned in the next decade are combining the capabilities of experimental tools highlighted in this communication in novel and ingenious ways, for instance, cryogenic ion beam storage devices with advanced light sources or ion accelerator facilities.

An exciting future lies ahead for those studying the gas phase dynamics of molecules. A key challenge for the community is to ease the transfer of knowledge between different fields whenever possible. Strides to achieve this are constantly being made and are aided by a range of successful international and interdisciplinary networks supported by, e.g., EU framework programs for research and innovation.
